# Whole-genome sequence of a human adenovirus species E strain identified from Kerala, India

**DOI:** 10.1128/mra.00527-26

**Published:** 2026-05-18

**Authors:** Balakrishnan Anukumar, Deepthi Kappala, Santosh M. Jadhav, Sruthi Nhavilthodi, Amalmol Peter, Sreelekha Kariyil Purushothaman

**Affiliations:** 1 ICMR—National Institute of Virology Kerala unit 680882 , Alappuzha, India; 2 ICMR—National Institute of Virology 29620, Pune, India; Queens College, Queens, New York, USA

**Keywords:** human adenovirus, HAdV-E4, whole-genome sequencing, Illumina, viral genomics, molecular surveillance, Kerala, India

## Abstract

We report the coding complete sequence of a human adenovirus species E type 4 (HAdV-E4) strain detected in a conjunctival specimen collected in Kerala, India in 2014. Illumina-based sequencing generated a 35,935 bp genome with a guanine-cytosine (GC) content of 56.4%, expanding the limited genomic data on HAdV-E circulating in India.

## ANNOUNCEMENT

Human adenoviruses are double-stranded DNA viruses that fall under the *Adenoviridae* family, *Mastadenovirus* genus, and *Human mastadenovirus* (HAdV) species and linked to respiratory infections, conjunctivitis, and gastroenteritis (1, 2). Among these, HAdV-E is unique because it includes only one recognized type, adenovirus type 4 (HAdV-4) (3), and has been identified as the cause of acute respiratory disease outbreaks, particularly in military settings (4–7).

This study details the coding complete sequence of a human adenovirus species E strain from Kerala, India. The sample was collected from a conjunctival swab of a 42-year-old man in Kottayam District, Kerala in 2014. The study was approved by the Institutional Human Ethics Committee of the ICMR-National Institute of Virology (NIV/IEC/2019/March/D-6-52).

The virus was isolated from conjunctival swab specimens placed in viral transport medium by inoculating onto confluent monolayers of human embryonic kidney 293 (HEK-293) cells. These cells were incubated until a cytopathic effect was observed (8). The culture supernatant was subsequently collected and stored at −80°C for later use.

The QIAamp Viral DNA Mini Kit (Qiagen, Hilden, Germany) was utilized to extract viral DNA. Viral DNA presence was confirmed using real-time PCR targeting the hexon gene (9). Sequencing libraries were constructed using the Illumina Viral Surveillance Panel v2 Kit, and then sequenced on an Illumina MiSeq platform with the MiSeq Reagent Kit v3 (600 cycles).

Initially, a total of 1,966,920 raw reads were generated before being subjected to data quality filtering. These reads were processed using fastp v0.23.2 (10) (minimum Phred score Q20, minimum length of 50 bp). After filtering, 1,954,688 reads remained, with 96.47% achieving Q20 and 89.71% reaching Q30 base pairs. The reads were aligned to the reference genome NC_003266.2 using BWA-MEM2 v2.2.1 (11), and the alignments were processed with Samtools v1.16 (12). Of these, 1,334,113 reads (68.25%) were mapped.Variants were identified using Bcftools v1.17(12) (minimum quality score 30, depth ≥10), and a consensus genome was created. Genome annotation was done using prokka v1.15.6 (13), which identified 36 protein-coding genes. For all tools, default parameters were used throughout the study unless otherwise specified. The resulting genome was 35,935 bp in length, with a guanine-cytosine (GC) content of 56.4% and an average depth of 5,025×. Since the terminal inverted repeat regions were not experimentally verified, the genome was labeled as coding complete.

**Fig 1:**
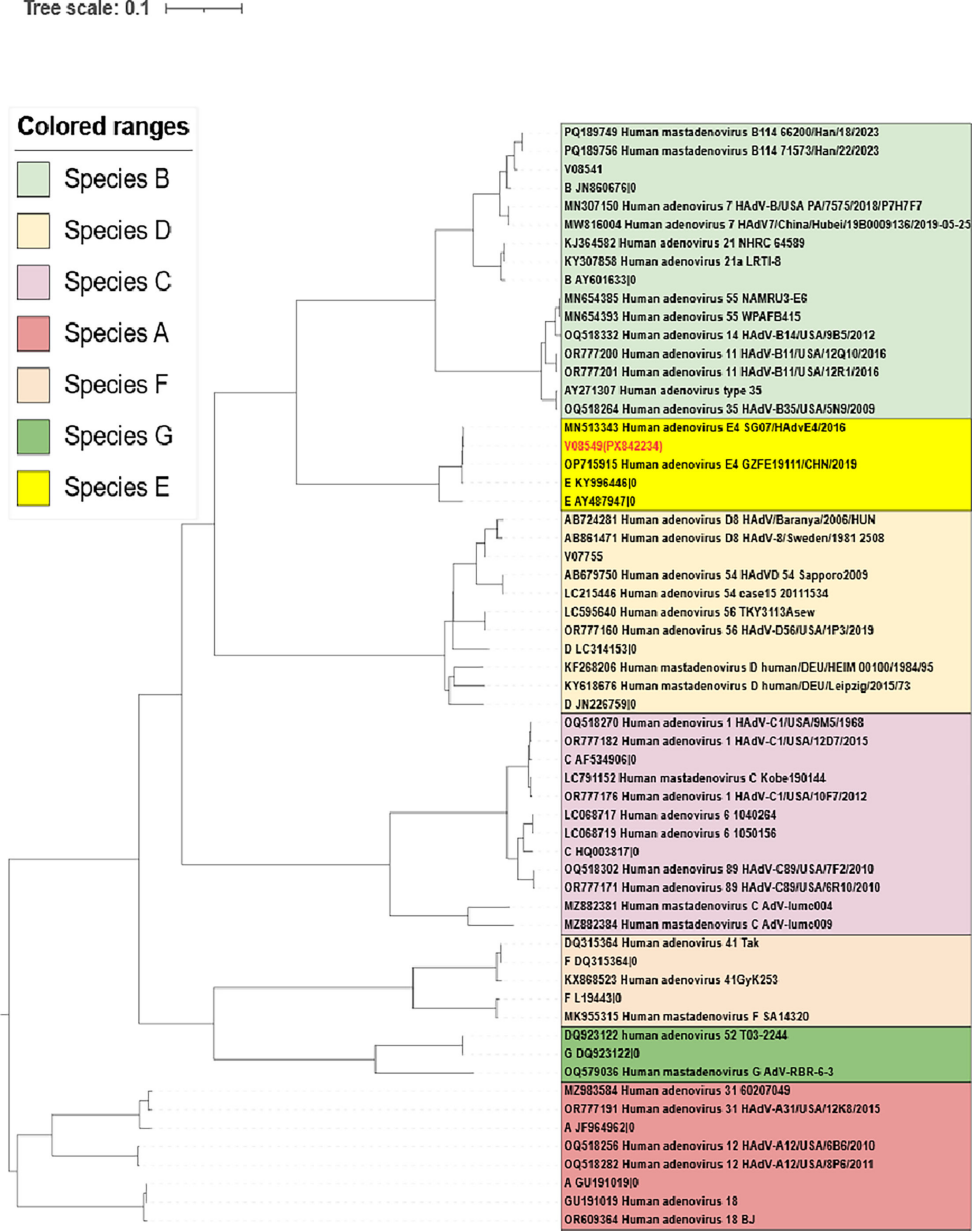
Maximum likelihood phylogenetic tree based on 60 whole-genome representative sequences of adenoviruses retrieved from the NCBI GenBank database. Multiple sequence alignment was performed using MAFFT v7.490, and the tree was constructed using MEGA v6.0 with the maximum composite likelihood method and visualized using iTOL. v7. The Kerala isolate PX842234 (V08549) clusters within the human adenovirus species E and groups closely with strain MN513343 from Singapore (2016).

For the phylogenetic analysis, 60 representative nucleotide sequences were selected from the National Center for Biotechnology Information (NCBI) GenBank database considering factors, such as geographic diversity, the year of isolation, and the completeness of the sequences. The complete genome sequences were aligned using MAFFT (v7.490) (14). Evolutionary distances were determined through the Maximum Composite Likelihood method (15), and a phylogenetic tree was generated with MEGA v6.0 (16) and subsequently edited using iTOL v7 (17). The Kerala isolate was found to be closely related to the Singapore strain (MN513343) from 2016 (Fig. 1).

This coding complete genome sequence contributes valuable data to the currently limited collection of HAdV-E genomes from India.

## Data Availability

GenBank accession number: PX842234. Sequence Read Archive (SRA) accession number: PRJNA1427409.
